# Intake of S-Methylmethionine Alters Glucose Metabolism and Hepatic Gene Expression in C57BL/6J High-Fat-Fed Mice

**DOI:** 10.3390/foods14010034

**Published:** 2024-12-26

**Authors:** Mariana Buranelo Egea, Gavin Pierce, Neil Shay

**Affiliations:** 1 Campus Rio Verde, Goiano Federal Institute of Education, Science and Technology, Rio Verde 75901-970, GO, Brazil; 2 Department of Food Science and Technology, Oregon State University, Corvallis, OR 97331, USA; gavinpierce42@gmail.com

**Keywords:** vitamin U, metabolic syndrome, glucose profile

## Abstract

A diet containing foods that are sources of S-methylmethionine (SMM), and its use as a dietary supplement, have demonstrated beneficial health effects. Thus, the objective of this work was to evaluate the inclusion of SMM as a dietary supplement in C57BL/6J high-fat-fed mice to verify whether this compound alone would be responsible for these positive effects. Mice were divided into three groups: LF (low-fat diet), HF (high-fat diet), and HF+SMM (high-fat diet plus SMM), and maintained for 10 weeks with water and food provided ad libitum. Body weight and food intake were measured weekly, and food efficiency was calculated. In addition, at week 9, fasting glucose was measured and, after necropsy, at week 10, liver, inguinal adipose, and kidney weights were measured; triglycerides, histology, liver gene expression, serum insulin, and MCP-1 levels were also determined. Final body weight, average weight gain, and the liver/body weight of the SMM group showed a significant difference with the LF group. HF+SMM-fed mice show improved regulation in glucose metabolism, demonstrated by the assessment of fasting glucose, insulin concentration, and HOMA-IR, compared with the HF-fed group. Liver triglycerides and MCP-1 levels showed no significant differences between fed groups. By the positive gene regulation of *Sult1e1*, *Phlda1*, and *Ciart*, we hypothesized that SMM administration to mice may have regulated xenobiotic, glucose, and circadian rhythm pathways.

## 1. Introduction

The Western diet, which is low in fruits and vegetables and high in fatty and sugary foods, has been considered a major contributor to the global obesity epidemic. Obesity is a chronic disease characterized by an increase in body mass, which occurs when more calories are consumed than expended. This situation has worsened with the occurrence of the COVID-19 pandemic, as some individuals experienced reduced their physical activity [1,2].

The presence of obesity in human health results in outcomes such as a higher risk of developing diseases such as type 2 diabetes, polyscystic ovarian syndrome, and cancers, among others. This is because obesity is considered to be a pro-inflammatory disease, which increases the number of natural killer cells, as well as a regulator of pro-inflammatory macrophages, which, in turn, causes a high production of pro-inflammatory cytokines and, with that, an imbalance in the antioxidant system [3,4]. In this sense, the concept of metabolic syndrome arises, which is defined as the occurrence of at least three of these five conditions: diabetes, overweight and obesity, triglyceridemia, hypercholesterolemia, and hypertension [5]. Since the occurrence of these diseases alters normal metabolism, they are called metabolic diseases; because the metabolism process is not regulated properly, the result is unbalanced energy production profiles [6]. In this sense, steatotic liver disease associated with metabolic dysfunction (MASLD) is characterized by the presence of hepatic steatosis and at least one of the five cardiometabolic risk factors, such as hypertension, pre-diabetes or diabetes, hypertriglyceridemia, and low-density lipoprotein (LDL-c) [7,8].

Regardless of the nomenclature used, it is known that, allied to practice of physical activity and quality of life, bioactive compounds, such as phenolic compounds, carotenoids, and amino acids, present in fruits and vegetables and can attenuate the inflammation caused by obesity and seem to promote improvement in the metabolism and biochemical aspects of the disease, improving MetS and MASD conditions [9].

S-Methylmethionine (SMM) is a derivative of L-methionine catabolism, which is first methylated to S-adenosylmethionine (SAM) and, then, the adenosyl group is replaced by a methyl group catalyzed by the enzyme methionine S-methyltransferase, making this molecule a potent methyl donor [10]. At normal concentrations in the liver, SAM directs homocysteine (a product of SAM catabolism) toward remethylation, using BHMT (homocysteine betaine methyltransferase) and betaine obtained from diet or transsulfuration (toxic homocysteine catabolism) to reestablish homeostasis [11].

In addition, SAM can be a methionine precursor that is involved in several functions in the body, including methylation reactions performed by methyltransferases for various molecules, such as proteins, nucleotides in DNA and RNA, and carbohydrates, among others, which affect a large number of biological processes [12]. At low concentrations of methionine, the content of SAM is also reduced and there is no inhibition of the enzymes BHMT and MTHFR (methylenetetrahydrofolatorreductase), which act in the remethylation pathway, that is, the synthesis of methionine from homocysteine. Studies demonstrate that low concentrations of SAM and glutathione (GSH) levels may result in reduced methylation levels, the alteration of gene expression, and the accumulation of oncogene expression, which alters DNA conformation and chromatin structure, as well as increased body mass and increased susceptibility to liver damage [12,13,14].

The administration of SMM has demonstrated antitumor effects against liver cancer and antioxidant activity [15], prevention of liver damage caused by valproic acid [16], protection against keratinocyte progenitor cells and human dermal fibroblasts (hDFs) from ultraviolet B irradiation (UVB) irradiation, and the reduction of UVB-induced skin erythema and immune suppression [17]. SMM presence has been reported for several foods such as tomato, cabbage, beetroot, and celery [18], as well as *Camellia sinensis* [19] and oolong [20] teas, among others. The ingestion of foods containing SMM or SMM supplementation increases hepatic SMM concentrations, and has been shown to improve the biochemical and metabolic parameters related to obesity [21,22].

Previous studies by our group demonstrated, by metabolomic analysis in mice, an increase in the hepatic content of SMM in the administration of raspberries [22], green tea [21], and watermelon and its by-products [23] in combination with a high-fat diet The increase in hepatic SMM was related to positive effects found on the physiological and metabolic parameters of these animals, such as a beneficial effect on the host microbiome [21] and the reduction of serum cholesterol [23]. In this work, we hypothesized that providing SMM as a dietary supplement could alleviate detrimental metabolic outcomes in C57BL/6J mice fed with a high-fat diet.

## 2. Materials and Methods

### 2.1. Mouse Diet Studies

Two groups of male C57BL/6J mice (Jackson Laboratory) (*n* = 12 per group) served as two groups of controls and were provided with either a low-fat diet (10% kcal fat) or a high-fat diet (45% kcal fat, 20% kcal sucrose, and 1% (*w/w*) cholesterol). The experimental group (*n* = 8 mice) was fed a high-fat diet supplemented with 1% (*w/w*) DL-methionine methylsulfonium chloride (SMM, Supplementary Table S1). All diets were formulated by Research Diets (New Brunswick, NJ, USA) and administered for a period of 10 weeks.

The mice were housed in groups of four per cage in a room maintained at 22 ± 2 °C with a 12 h light/dark cycle, with ad libitum access to food and distilled water throughout the study. Diet consumption was measured weekly from weeks 4 to 8 (*n* = 5 weeks) for each group. Food efficiency was calculated for each week of the study (*n* = 10 weeks) as the ratio of average weekly body weight gain to total energy intake.

At the end of the study, mice were fasted for 5 h, anesthetized, and euthanized by cervical dislocation. Blood samples were obtained through cardiac puncture, allowed to incubate on ice for 60 min, and then centrifuged at 1000× *g* for 15 min at 4 °C. The resulting serum was collected and stored at −80 °C. Liver, inguinal adipose tissue, and kidneys were harvested and weighed. A 100 mg sample of liver tissue was preserved in 10% buffered formalin for histological examination, and the remaining tissues were rapidly frozen on dry ice and stored at −80 °C. All animal procedures adhered to the Guidelines for Care and Use of Laboratory Animals set by Oregon State University and were approved by the Oregon State University Animal Care and Use Committee (ACUP Protocol #5107).

### 2.2. Fasting Glucose

In week 9, fasting blood glucose levels were measured, as described by Egea et al. [24]. Briefly, after fasting for 4 h, mice were sedated with isoflurane, held in flat bottomed rodent-restraining tubes, and a small tail incision was made; fasting blood glucose was measured using a handheld glucometer (Contour Next EZ, Bayer Healthcare LLC, Mashawaka, IN, USA). The cuts were disinfected with 70% ethanol wipes before and after each blood glucose measurement.

### 2.3. Hepatic Histological and Total Triglycerides Analysis

Liver tissue was preserved in 10% buffered formalin and embedded in paraffin. Two 5-μm sections from each liver sample were placed on numbered slides and subsequently stained with hematoxylin-eosin (Nationwide Histology, Spokane, WA, USA). Images were captured using an Olympus IX71 light microscope (Olympus, PA, USA).

Liver tissue (~100 g) was homogenized in NP40 Substitute Assay Reagent (Cayman Chemicals, Ann Arbor, MI, USA). The homogenates were centrifuged for 10 min at 10,000× *g* at 4 °C, and the supernatants were diluted 1:10 with NP40 Substitute Assay Reagent. Triglyceride levels were measured using a colorimetric assay kit (reference 10010303; Cayman Chemicals).

### 2.4. Serum ELISA

Serum insulin and MCP-1 (Monocyte Chemoattractant Protein-1) concentrations were quantified in 96-well plates using enzyme-linked immunosorbent assay (ELISA) kits according to the manufacturer’s guidelines (Insulin kit, Thermo Scientific, Frederick, MD, USA; MCP-1 ELISA kit, Invitrogen Corporation, Camarillo, CA, USA). Once each assay was completed, the plates were analyzed using a Luminex 200 instrument and xPONENT version 4.0 software.

### 2.5. Homeostasis Model Assessment

A Homeostatic Model Assessment for Insulin Resistance (HOMA-IR) and Homeostatic Model Assessment for function of pancreatic beta cells (HOMA-%B) were calculated following the method of Heikkinen et al. [25].

### 2.6. RNA Sequencing

At necropsy, liver tissue was stored in RNA later, and stored at −20 °C. Liver tissue (~50 mg) was then homogenized and RNA was isolated using the Trizol reagent (Invitrogen, Carlsbad, CA, USA), according to standard procedure. Isolated RNA was analyzed for purity with a Nano Drop 2000 spectrophotometer (Thermo Scientific, Carlsbad, CA, USA) at λ260/280 and λ260/230 score, and three samples of each group with the highest RIN numbers were used for RNAseq. Sequencing libraries were generated using the NEBNext^®^ Ultra™ RNA Library Prep Kit for Illumina^®^ (NEB, Ipswich, MA, USA), following the manufacturer’s standard protocols. The six constructed mRNA libraries were sequenced on an Illumina HiSeq 2000 (Illumina, San Diego, CA, USA) at Novogene Technology Co., Ltd. (Novogene Gene Technology, Davis, CA, USA). RNA sequencing and analysis were performed as described previously [24]. Global gene expression was compared between the SMM, LF, and HF groups.

### 2.7. Statistical Analysis

Data are expressed as mean ± SEM. Statistical analysis was performed using one-way ANOVA, and post hoc comparisons were conducted with Tukey’s test. Significance was set at *p* < 0.05, while a trend toward significance was considered for 0.05 < *p* < 0.10. All analyses were performed using GraphPad Prism 6 software (version 6.0, GraphPad Software, San Diego, CA, USA).

## 3. Results

### 3.1. Energy Intake and Weight Gain

**Figure 1:**
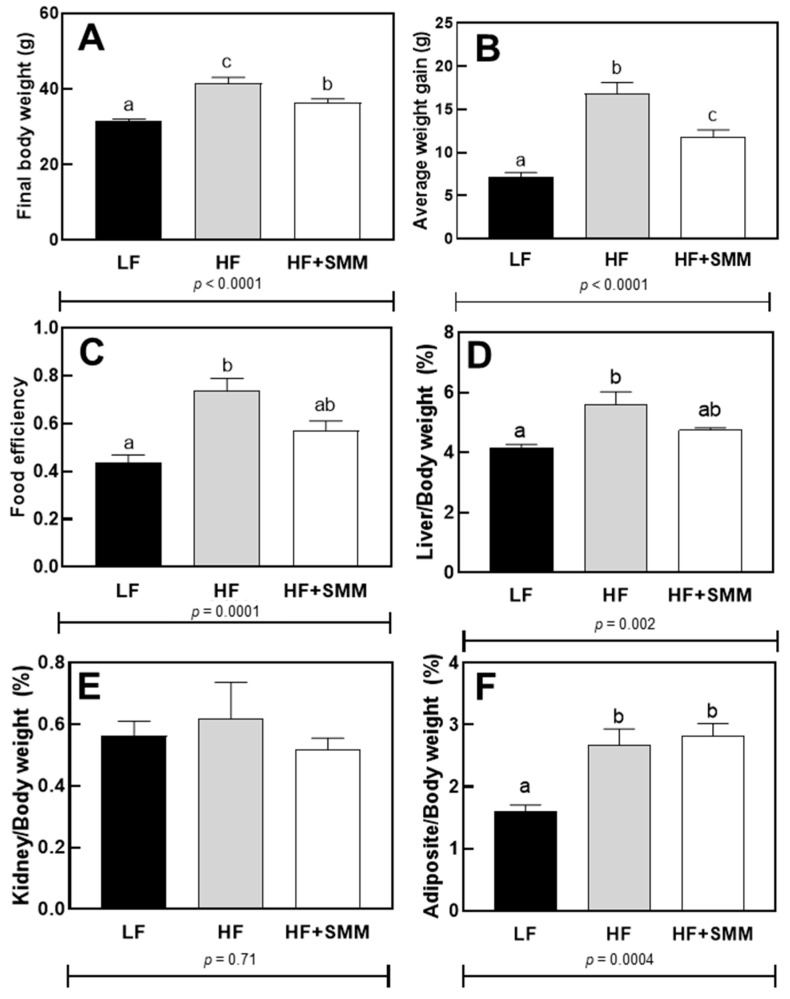
Final body weight (**A**), Average weight gain (**B**), Food efficiency (**C**), Liver /body weight (**D**), Kidney/body weight (**E**), Adipose/body weight (**F**) in male C57BL/6J mice fed either a low-fat (LF) diet, a high-fat (HF) diet, or HF plus DL-methionine methylsulfonium chloride (HF+SMM) after 10 weeks. Groups not sharing the same lowercase letters indicate that one-way ANOVA found significant differences between groups (*p* < 0.05). Values shown are the average (*n* = 12 for control groups and *n* = eight for the experimental group) ± SEM.

The body weight (final and gain weight) (Figure 1A,B) of the mice showed a significant difference between all groups (*p* < 0.0001). The food efficiency (Figure 1C) of HF+SMM group was not different from the LFgroup and HF fed groups, but the LF and HF groups were different from each other (*p* < 0.0001).

Ratios of kidney, liver, and intraperitoneal adipose weights to total body weight are shown in Figure 1C,E,F, respectively. Ratios for the HF+SMM group was not different from the control groups (LF- and HF-fed groups) (*p* < 0.002) for liver/body weight ratio, while HF+SMM diet increased adipose/body weight ratio compared to the LF-fed group (*p* < 0.0004). No significant difference was demonstrated in the kidney weight/total body ratio.

### 3.2. Biochemical Parameters

**Figure 2:**
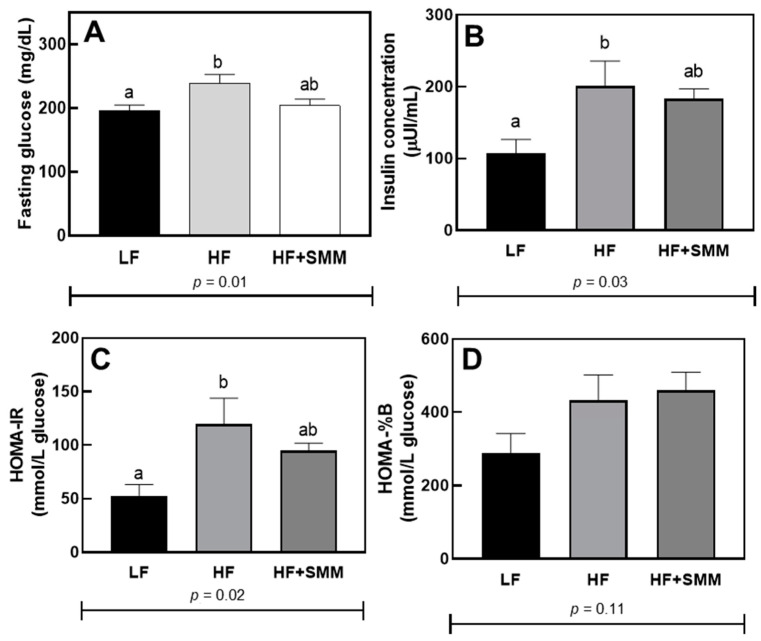
Fasting glucose at week 9 (**A**), serum insulin concentration (**B**), homeostasis model assessment of insulin resistance (HOMA-IR) (**C**), and homeostatic model assessment of β-cell function (HOMA-%B) (**D**) in male C57BL/6J mice fed either a low fat (LF) diet, a high fat (HF) diet, and HF plus DL-methionine methylsulfonium chloride (HF+SMM) after 10 weeks. Groups not sharing the same lowercase letters indicate that one-way ANOVA found significant differences between groups (*p* < 0.05). Values shown are averages (*n* = 12 for control groups and *n* = 8 for the experimental group) ± SEM.

Figure 2 shows the results obtained from the baseline glucose and insulin measurements, taken at week 9, as well as the homeostasis model assessment of insulin resistance (HOMA-IR) and the homeostatic model assessment of β-cell function (HOMA-%B) calculated from these values. For fasting glucose, insulin concentration, and HOMA-IR, the HF-fed group was significantly higher than LF-fed group, and HF+SMM-fed mice showed no difference with LF-fed mice (*p* < 0.01, 0.03, and 0.03, respectively). No significant difference was demonstrated for HOMA-%B value among any groups.

**Figure 3:**
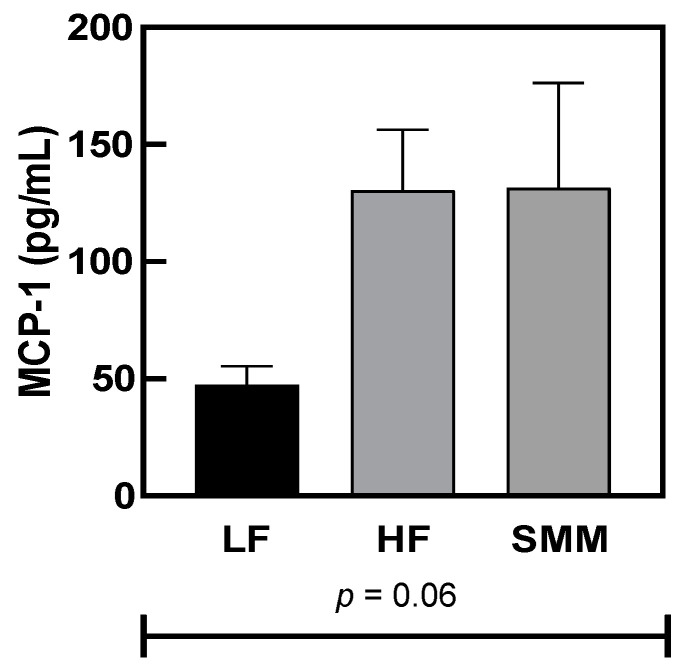
Serum Monocyte Chemoattractant Protein-1 (MCP-1) concentration (B) in male C57BL/6J mice fed either a low-fat (LF) diet, a high-fat (HF) diet, or HF plus DL-methionine methylsulfonium chloride (HF+SMM) after 10 weeks. One-way ANOVA indicated no significant differences between diet groups (*p* < 0.05). Values shown are averages (*n* = 12 for control groups and *n* = 8 for the experimental group) ± SEM.

Figure 3 shows the serum MCP-1 concentration in all fed groups. No significant difference was demonstrated for serum MCP-1 concentration for all studied groups.

**Figure 4:**
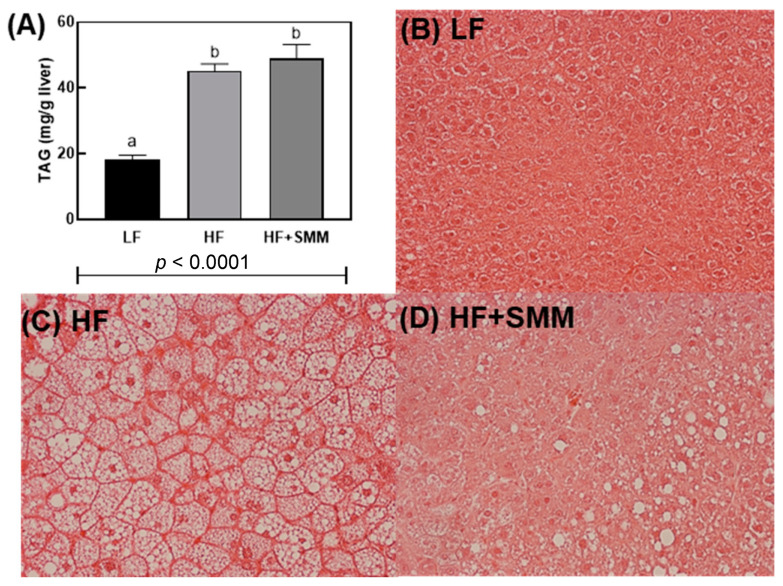
Total triacylglycerol (TAG) assay (**A**) and a study of hematoxylin-eosin-stained liver cross-sections of male C57BL/6J mice fed a LF diet (**B**), a HF diet (**C**), and HF+SMM (**D**) for 10 weeks. Slides were observed under 400 magnification (40× objective), using an Olympus IX71 light microscope (Olympus, PA, USA). (a,b) Groups not sharing the same lowercase letters indicate that one-way ANOVA found significant differences between groups (*p* < 0.05). Average (*n* = 12 for control groups and *n* = 8 for the experimental group) ± SEM.

Total triacylglycerol concentration (TAG) was statistically higher in HF- and HF+SMM-fed groups vs. LF-fed mice (*p* < 0.0001) (Figure 4A). This is confirmed by visual inspection of corresponding microphotographs of the stained liver tissue (Figure 4B–D).

### 3.3. Gene Expression

Supplementary Figure S1 presents the quality control data from the Transcriptome Analysis Console (TAC) Software for the RNAseq of liver tissue in male C57BL/6J mice that were fed either a LF, HF, or HF+SMM diet for 10 weeks. Principal component analysis showed that axis 1 accounted for 19.9%, axis 2 for 15.6%, and axis 3 for 11.1% of the total variance in the original dataset (A). The data quality was considered satisfactory when compared to hybridization controls and target preparation controls (B and C). The probe set (D) and box plot (E) demonstrated clear separation between positive and negative controls.

**Figure 5:**
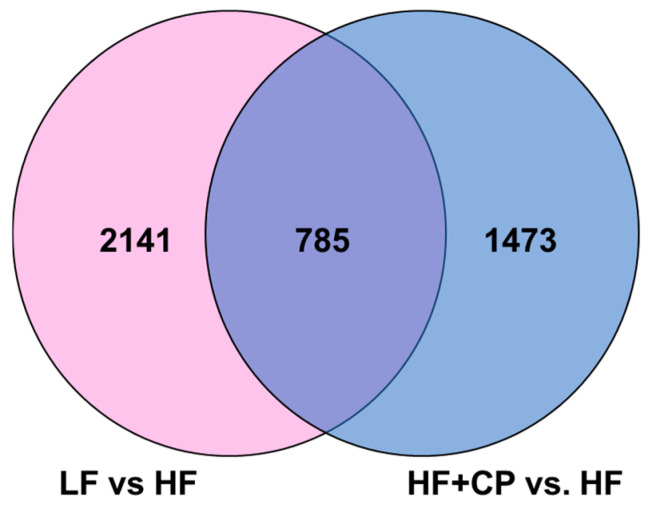
Venn diagram with gene expression of liver of male C57BL/6J mice fed either a LF diet or HF+SMM diet.

A total of 5520 distinct transcripts were examined (Figure 5). Of these, 4399 were differentially expressed (*p* < 0.05), with 2141 genes uniquely expressed in the LF- vs. HF-fed groups, 1473 genes uniquely expressed in the HF+CP- vs. HF-fed groups; 785 genes expressed in both LF- vs. HF- and HF+CP- vs. HF-fed groups.

The 25 canonical pathways (Table 1) most impacted by the HF+SMM group compared to the HF group are shown in Table 1. Even without demonstrating a significant change in lipid metabolism (Figure 4A), it was possible to notice that several pathways were regulated (Table 1) in the administration of SMM to mice, among the following: Fatty acid beta-oxidation (3&6), Steroid biosynthesis (5), Nuclear receptors in lipid metabolism and toxicity (7), Statin pathway (8), Omega-9 fatty acid synthesis (13), Cholesterol metabolism with Bloch, Kandutsch–Russell pathways (18), P450 monooxygenases (21), and SREBF and miR33 in cholesterol and lipid homeostasis (24).

Table 2 presents the 25 most downregulated and upregulated genes in liver tissue. For HF+SMM vs. HF, it was apparent that many pathways related to lipid metabolism and regulation, xenobiotic mechanism, and circadian rhythm, which is consistent with the canonical pathways reported in Table 1.

## 4. Discussion

In the present study, the final body weight, average weight gain, and food efficiency (Figure 1A–C) of the SMM group showed a significant difference with the LF group. The values demonstrated for the SMM group were also lower than those demonstrated for the HF control group. This reduction of weight change with the administration of a high-fat diet may be associated with a lower number of oil droplets in the liver tissue (Figure 4B–D), corroborating what had been reported by Lee et al. [26], who studied SMM treated cells and compared them with untreated cells and reported this behavior.

The SMM treatment-regulated glucose metabolism of the SMM-group, demonstrated by the assessment of fasting glucose, insulin concentration, and HOMA-IR (Figure 2A–C, respectively), showed no significant difference with the LF group. This behavior may be related to the positive aspects found in this study related to body mass. It is known that the increase in body mass is associated with factors such as changes in glucose levels and glucose tolerance, as well as developments in insulin resistance [27].

Even with the positive results in glucose metabolism and physical parameters, in the present study, no differences were found in TAG levels between the group treated with HF+SMM and the HF group (Figure 4A), demonstrating that, although the SMM administration has altered the glucose parameters of the animals, TAG accumulation was not significantly altered. Mezei et al. [28] suggested that the decrease in serum TGA may not be directly related to a decrease in body weight, as was also observed in the present study. This literature statement corroborates our present work, considering the number of canonical pathways that were activated in the liver with SMM administration (9/25 canonical pathways, Table 1). Furthermore, Lee et al. [26] demonstrated that SMM treatment also resulted in a decrease in intracellular TAG levels that could be related to an inhibition of adipocyte differentiation. This inhibition may not have occurred in the present study, since there was no difference between adiposity tissue/body weight ratio in the SMM group and the HF group (Figure 1F).

Regarding the canonical pathways and regulation of gene expression, it is evident that the PPAR or peroxisome proliferator-activated receptor-signaling pathway was regulated by SMM administration (Table 1). PPARs are involved in the transcription of gene-encoding enzymes related to lipid metabolism and adipocyte differentiation [28]. In addition, the activation of PPARs is also related to greater insulin sensitivity [29], corroborating the positive results found in the present study regarding glucose metabolism (Figure 2).

A high-calorie diet in mice alters patterns of activity, feeding, and hormone production during the circadian rhythm length [30,31]. When mice are fed with a high-fat diet, the animals have a pattern of change in the length of the circadian rhythm that results in a change in feeding, which normally occurs largely during nighttime, to daytime and in turn to greater body weight [32]. These alterations have been related to hepatic gene expression and the gene expression of *Ciart* and *Per3*, genes involved with the circadian rhythm, and have been inversely related to the presence of plasma lipids and body weight [32,33]. In our results, the circadian rhythm seems to have been regulated in the SMM-group mice, since *Ciart* and *Per3* were upregulated (4.71- and 2.54-fold change, respectively), and this may have contributed to the positive results found for body weight (Figure 1), since that regulation of this pathway can result in homeostasis considering food consumption and energy expenditure. This behavior still needs to be confirmed in humans, but our research suggests that there may be a possible positive effect related to the regulation of the circadian rhythm in individuals with obesity after the continuous ingestion of SMM.

In the present work, a large number of genes that were upregulated in liver tissue were shown to be responsible for cell growth and differentiation, such as *Foxq1* (11.64-fold change), *Myc* (10.06-fold change), *Gadd45g* (5.68-fold change), *Wee1* (4.48-fold change change), *C6* (2.97-fold change), *Dnase1l3* (2.79-fold change), and *Capn8* (2.55-fold change). Previous results have shown the that treatment of hepatocytes with SMM prevents liver damage by increasing enzymatic antioxidant activity and the proliferation of hepatocytes [16].

Furthermore, the liver is a vital organ where the detoxification of xenobiotic substances occurs and which acts when the accumulation of fat in the liver occurs [34]. In the present work, gene expression related to xenobiotic metabolism was demonstrated by the presence of genes such as *Sult1e1* and *Marco* (38.1- and 3.94-fold change, respectively), which were upregulated. Turkyilmaz & Yanardag [35] have shown that the SMM administration ameliorated liver injury of Male Sprague–Dawley rats by decreasing ˠ-GT (an enzyme that catalyzes the breakdown of glutathione). With the presence of glutathione, a protein that is part of the endogenous antioxidant system, hepatocytes may be protected from the action of free radicals from the inflammatory process caused by the development of obesity [36]. These are an important findings because the enzymatic antioxidant activity of the human body plays an important role in MASLD, especially decreasing the progression of steatosis, steatohepatitis, cirrhosis, and, finally, cancer [37].

In closing, we report here that, in addition to exerting an hypolipidemic effect as reported by other authors, SMM administration demonstrated potential changes in circadian rhythm and other pathways. All studies, in in vivo, of animal models have limitations, and this study is no different. All inferences made in this study need to be confirmed in randomized controlled trials in humans so that the effective dosage and duration of action can be established for use as therapy in patients living with obesity.

## 5. Conclusions

SMM administration reduced the final body weight and average weight gain of C57BL/6J high-fat-fed mice compared with high-fat-group. Meanwhile, for food efficiency, liver weight/body weight, fasting glucose, serum insulin concentration, and homeostasis model assessment of insulin resistance, the administration of this supplement showed no difference with the LF group. Furthermore, the evaluation of gene regulation demonstrated that the xenobiotic, glucose and circadian rhythm pathways also appear to be regulated by the administration of SMM.

## Figures and Tables

**Table 1:** Canonical pathways differentially regulated in the liver of male C57BL/6J mice fed either HF or HF+SMM.

	Pathway	#Total	#Up	#Down	Significance	*p*-Value
1	PPAR signaling pathway	20	10	10	3.77	1.71 × 10^−4^
2	G protein-coupled receptor	7	3	4	3.49	3.22 × 10^−4^
3	Fatty acid beta-oxidation (streamlined)	10	4	6	3.02	9.46 × 10^−4^
4	Exercise-induced circadian regulation	13	5	8	3.01	9.83 × 10^−4^
5	Steroid biosynthesis	6	5	1	2.99	1.03 × 10^−3^
6	Fatty acid beta-oxidation	10	2	8	2.69	2.03 × 10^−3^
7	Nuclear receptors in lipid metabolism and toxicity	9	7	2	2.63	2.32 × 10^−3^
8	Statin pathway	7	5	2	2.57	2.68 × 10^−3^
9	Mapk cascade	8	6	2	2.15	7.06 × 10^−3^
10	Glucocorticoid and mineralcorticoid metabolism	5	5	0	2.15	7.06 × 10^−3^
11	Protein–protein interactions in podocytes	109	50	59	2.05	8.83 × 10^−3^
12	mRNA processing	32	13	19	2.04	9.20 × 10^−3^
13	Omega-9 fatty acid synthesis	5	2	3	2	1.01 × 10^−2^
14	Endochondral ossification	13	6	7	1.99	1.01 × 10^−2^
15	GPCRs, non-odorant	15	9	6	1.97	1.07 × 10^−2^
16	GPCRs, odorant	12	9	3	1.96	1.09 × 10^−2^
17	Nuclear receptors	9	5	4	1.9	1.26 × 10^−2^
18	Cholesterol metabolism with Bloch and Kandutsch–Russell pathways	12	7	5	1.78	1.66 × 10^−2^
19	Protein–protein interactions in the podocyte	45	20	25	1.72	1.92 × 10^−2^
20	ESC pluripotency pathways	20	12	8	1.67	2.14 × 10^−2^
21	Eicosanoid metabolism via cytochrome P450 monooxygenases	5	5	0	1.62	2.40 × 10^−2^
22	Mapk signaling pathway	26	14	12	1.52	3.02 × 10^−2^
23	Purine metabolism	27	20	7	1.47	3.42 × 10^−2^
24	SREBF and miR33 in cholesterol and lipid homeostasis	4	3	1	1.44	3.65 × 10^−2^
25	One-carbon metabolism and related pathways	10	6	4	1.41	3.93 × 10^−2^

**Table 2:** Top 20 up and downregulated hepatic mRNAs from male C57BL/6J mice fed HF (high-fat) plus DL-methionine methylsulfonium chloride (SMM) vs. HF for 10 weeks.

	Upregulated				Downregulated			
	Fold Change	*p*-Value	Gene Symbol	Function	Fold Change	*p*-Value	Gene Symbol	Function
1	38.1	1.00 × 10^−4^	*Sult1e1*	Xenobiotic metabolism	−5.47	1.51 × 10^−2^	Tubb2a	Intracellular transport
2	11.64	3.20 × 10^−3^	*Foxq1*	Cellular growth	−3.93	1.11 × 10^−2^	*Csad*	Decarboxylation activity of aminoacids
3	10.06	5.00 × 10^−3^	*Myc*	Cellular growth	−3.65	4.92 × 10^−5^	*Spon2*	Lipid metabolism
4	7.03	2.00 × 10^−3^	*Phlda1*	Glucose metabolism	−3.63	1.00 × 10^−3^	*Cyr61*	Extracellular matrix
5	6.18	5.00 × 10^−4^	*Usp2*	Fatty acid metabolism	−3.62	1.30 × 10^−3^	*G0s2*	Lipid metabolism
6	5.68	3.58 × 10^−2^	*Gadd45g*	Cellular growth	−3.59	2.00 × 10^−4^	*Elovl3*	Fatty acid metabolism
7	4.92	3.00 × 10^−4^	*Obp2a*	Fatty acid metabolism	−3.38	1.56 × 10^−2^	*Ppp1r3c*	Glycogen hydrolysis
8	4.71	2.00 × 10^−4^	*Ciart*	Circadian rhythm	−3.3	7.00 × 10^−4^	*Arsg*	Lipid and glucose metabolism
9	4.48	1.00 × 10^−4^	*Wee1*	Cellular growth	−3.26	3.27 × 10^−2^	*Slco1a4*	Transport related gene
10	4.03	1.37 × 10^−2^	*Moxd1*	Oxidoreductase activity	−3.26	9.35 × 10^−5^	*Insig2*	Sterol regulatory element
11	3.94	1.56 × 10^−2^	*Marco*	Xenobiotic metabolism	−3.04	3.30 × 10^−3^	*Cyp39a1*	cholesterol, steroids, and other lipids metabolism
12	3.58	6.47 × 10^−5^	*Per1*	Circadian rhythm	−3.01	2.70 × 10^−3^	*Mtnr1a*	Circadian rhythm
13	3.43	6.70 × 10^−3^	*Plk3*	Regulates cell cycle progression	−2.89	7.80 × 10^−3^	*Anxa2*	Regulates celluar growth
14	3.25	1.49 × 10^−6^	*Por*	Fatty acid metabolism	−2.76	7.00 × 10^−4^	*Acly*	Acetyl-CoA synthesis
15	3.23	3.17 × 10^−5^	*Gpcpd1*	Glycerophospholipid biosynthesis	−2.63	4.00 × 10^−4^	*Gm4952*	Transfers the acyl group to the N-terminus of glycine.
16	3.21	2.34 × 10^−2^	*Alas1*	Catalyzes rate-limiting step in heme biosynthesis.	−2.59	1.10 × 10^−3^	*Dtx4*	Cell regulation
17	3.19	1.18 × 10^−5^	*Ehd3*	Cell regulation	−2.58	1.06 × 10^−2^	*Spp1*	cell growth and differentiation
18	2.97	4.00 × 10^−4^	*C6*	Immune response	−2.57	1.30 × 10^−3^	*S100a10*	cell growth and differentiation
19	2.94	1.96 × 10^−2^	*Thrsp*	Lipid metabolism	−2.54	2.00 × 10^−4^	*Leap2*	Xerobiotic efflux
20	2.79	3.00 × 10^−4^	*Dnase1l3*	Cell apoptosis	−2.49	2.00 × 10^−4^	*St5*	Cell differentiation
21	2.74	1.50 × 10^−3^	*Afmid*	NAD metabolism	−2.39	2.38 × 10^−2^	*Tkfc*	DNA replication
22	2.66	4.19 × 10^−2^	*Hsd3b4*	Biosynthesis of all classes of hormonal steroids	−2.35	1.00 × 10^−2^	*Inhbe*	Hormone regulation
23	2.62	3.00 × 10^−4^	*Fcgr2b*	Immune system	−2.27	8.00 × 10^−4^	*Psen2*	cell growth and differentiation
24	2.55	1.62 × 10^−2^	*Capn8*	Membrane trafficking	−2.24	1.10 × 10^−3^	*Xk*	Lipid transportation
25	2.54	3.50 × 10^−3^	*Per3*	Circadian rhythm	−2.22	2.00 × 10^−4^	*Gldc*	Amino acid degradation

## Data Availability

The original contributions presented in the study are included in the article/Supplementary Material, further inquiries can be directed to the corresponding authors.

## Supplementary Materials

The following supporting information can be downloaded at https://www.mdpi.com/article/10.3390/foods14010034/s1, Figure S1: Quality control (QC) from Transcriptome Analysis Console (TAC) Software for the RNA sequencing of liver tissue in male C57BL/6J mice fed either a low fat (LF) diet, a high fat (HF) diet, and HF plus DL-methionine methylsulfonium chloride (SMM) after 10 weeks, Table S1: Diet formulation to low fat (LF), high fat (HF), and high fat plus DL-methionine methylsulfonium chloride (HF+SMM). Rodent Diet with 45 kcal% Fat and 1% Cholesterol and Same With Added 1% DL-methionine methylsulfonium chloride.
