# Supplementary material for: Intake of S-Methylmethionine Alters Glucose Metabolism and Hepatic Gene Expression in C57BL/6J High-Fat-Fed Mice
Source: Foods. 2024 Dec 26;14(1):34. doi: 10.3390/foods14010034 (PMC11720019; doi:10.3390/foods14010034)

Supplementary Table 1. Diet formulation to low fat (LF), high fat (HF), and high fat plus DL-methionine methylsulfonium chloride (HF+SMM). Rodent Diet with 45 kcal% Fat and 1% Cholesterol and Same With Added 1% DL-methionine methylsulfonium chloride

**Product #**	**LF diet**	**HF diet**	**HF+SMM diet**
	10 kcal% Fat	*45 kcal% Fat with*	*1% S-Methylmethionine*
		1% Chol Control	
**Ingredient**	**gm**	**gm**	**gm**
Casein	200	200	200
L-Cystine	3	3	3
Corn Starch	452.2	72.8	72.8
Maltodextrin 10	75	100	100
Sucrose	172.8	172.8	172.8
Cellulose, BW200	50	50	50
Soybean Oil	25	25	25
Lard	20	177.5	177.5
Mineral Mix S10026	10	10	10
DiCalcium Phosphate	13	13	13
Calcium Carbonate	5.5	5.5	5.5
Potassium Citrate, 1 H2O	16.5	16.5	16.5
Vitamin Mix V10001	10	10	10
Choline Bitartrate	2	2	2
Cholesterol	0	8.5	8.5
S-Methylmethionine		0	8.8
FD&C Yellow Dye #5	0.04	0	0
FD&C Blue Dye #1		0	0.025
FD&C Red Dye #40	0.01	0	0.025
**Total**	**1055.05**	**866.6**	**875.45**
**g**	**D12450H**	**D09071604**	**D19020110**
Protein	177.0	177.0	177.0
Carbohydrate	710.0	355.6	355.6
Sugar	182.8	182.8	182.8
Starch	527.2	172.8	172.8
Fat	47.4	204.9	204.9
Fiber	50.0	50.0	50.0
**g%**			
Protein	16.8	20.4	20.2
Carbohydrate	67.3	41.0	40.6
Fat	4.5	23.6	23.4
Fiber	4.7	5.8	5.7
**S-Methylmethionine**		**0**	**1.0**

**kcal**			
Protein	*708*	*708*	*708*
Carbohydrate	*2840*	*1422*	*1422*
Fat	*427*	*1844*	*1844*
Total	*3975*	*3975*	*3975*
**kcal%**			
Protein	*18*	*18*	*18*
Carbohydrate	**71**	*36*	*36*
Fat	*11*	*46*	*46*
kcal / gm	**3.77**	**4.59**	**4.54**

Supplementary Figure 1. Quality control (QC) from Transcriptome Analysis Console (TAC) Software for the RNA sequencing of liver tissue in male C57BL/6J mice fed either a low fat (LF) diet, a high fat (HF) diet, and HF plus DL-methionine methylsulfonium chloride (SMM) after 10 weeks

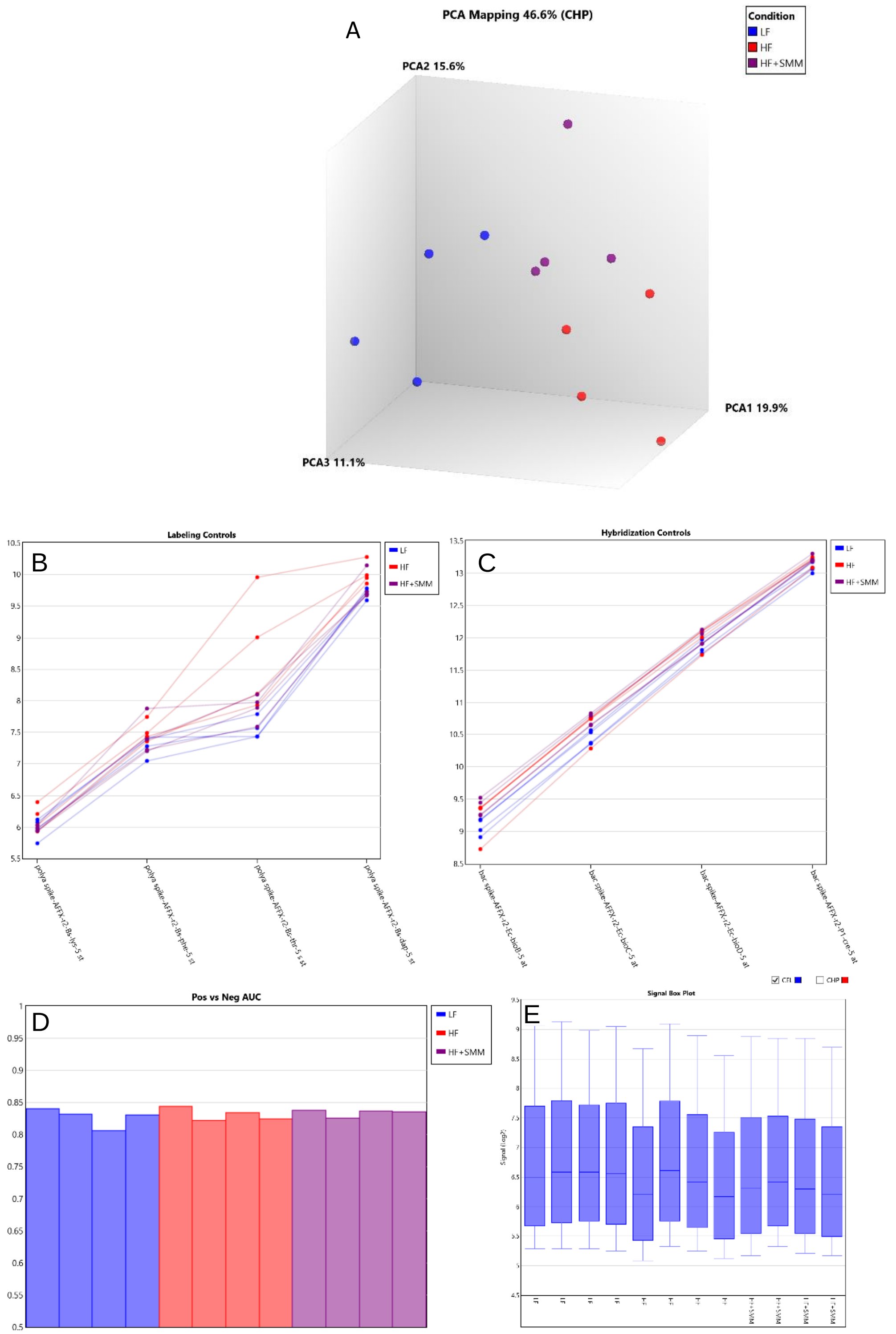

Supplement: Supplementary file 1 [file foods-14-00034-s001.zip › foods-3343699-supplementary.pdf]
